# Protective Effects and Mechanisms of Recombinant Human Glutathione Peroxidase 4 on Isoproterenol-Induced Myocardial Ischemia Injury

**DOI:** 10.1155/2021/6632813

**Published:** 2021-09-07

**Authors:** Chang Liu, Bozhao Li, Qi Yan, Shaopeng Niu, Yiding Zhao, Changhao Xiong, Tianjiao Zhang, Jingyan Wei

**Affiliations:** ^1^College of Pharmaceutical Science, Jilin University, Changchun 130021, China; ^2^College of Agriculture and Environmental Science, University of California Davis, Davis, CA 95616, USA; ^3^Key Laboratory for Molecular Enzymology and Engineering of the Ministry of Education, Jilin University, Changchun 130000, China

## Abstract

Ischemic heart disease (IHD) is a cardiovascular disease with high fatality rate, and its pathogenesis is closely related to oxidative stress. Reactive oxygen species (ROS) in oxidative stress can lead to myocardial ischemia (MI) injury in many ways. Therefore, the application of antioxidants may be an effective way to prevent IHD. In recent years, glutathione peroxidase 4 (GPx4) has received increasing attention due to its antioxidant effect. In a previous study, we used the new chimeric tRNA^UTuT6^ to express highly active recombinant human GPx4 (rhGPx4) in amber-less *Escherichia coli*. In this study, we established an isoproterenol- (ISO-) induced MI injury model in rats and an in vitro model to research the protective effect and mechanism of rhGPx4 on MI injury. The results showed that rhGPx4 could reduce the area of myocardial infarction and ameliorate the pathological injury of heart tissue, significantly reduce ISO-induced abnormalities on electrocardiogram (ECG) and cardiac serum biomarkers, protect mitochondrial function, and attenuate cardiac oxidative stress injury. In an in vitro model, the results also confirmed that rhGPx4 could inhibit ISO-induced oxidative stress injury and cardiomyocyte apoptosis. The mechanism of action of rhGPx4 involves not only the inhibition of lipid peroxidation by eliminating ROS but also keeping a normal level of endogenous antioxidant enzymes by eliminating ROS, thereby preventing oxidative stress injury in cardiomyocytes. Additionally, rhGPx4 could inhibit cardiomyocyte apoptosis through a mitochondria-dependent pathway. In short, rhGPx4, a recombinant antioxidant enzyme, can play an important role in the prevention of IHD and may have great potential for application.

## 1. Introduction

Ischemic heart disease (IHD) has been one of the leading causes of mortality in the world [1]. Myocardial ischemia (MI) is attributable to the reduction of blood perfusion in the heart, which leads to a decrease in the oxygen supply of the heart and disturbance of energy metabolism, resulting in the inability of the heart to work normally [2]. Oxidative stress is closely related to the occurrence and development of MI, and cardiomyocytes can produce a large amount of reactive oxygen species (ROS) under ischemia and hypoxia [3]. Excessive ROS can cause cardiac oxidative stress injury, leading to abnormalities in the myocardial structure and function [4, 5]. Therefore, the development of new antioxidants may provide new approaches for the prevention and treatment of IHD.

Glutathione peroxidase 4 (GPx4) is a selenium-containing antioxidant enzyme in monomeric form. It cannot only catalyze small molecular hydroperoxide but also reduce phospholipid hydroperoxide in the membrane and lipoprotein to inhibit lipid peroxidation [6, 7]. Many studies have identified that GPx4 is related to a variety of physiological and pathological processes, including sperm production, growth and development, endothelial dysfunction, neurodegenerative diseases, and oxidative stress injury [8–11]. Therefore, GPx4 has potential application value. In previous research, we successfully developed a new chimeric tRNA^UTuT6^ method to efficiently express glutathione peroxidase (GPx) in amber-less *Escherichia coli* (*E. coli*) based on the work of the Dieter Söll group at Yale University [12]. The recombinant human GPx4 (rhGPx4) expressed by this method has high activity and the structure of natural GPx4 [13]. Therefore, rhGPx4 is an ideal recombinant antioxidant enzyme.

Isoproterenol- (ISO-) induced MI injury in rats is a commonly used model for evaluating anti-MI drugs. In this model, large doses of ISO cause myocardial ischemia and hypoxia. Then, the generation and accumulation of ROS lead to oxidative stress injury in cardiomyocytes [14–18]. In this study, we established an ISO-induced MI injury model in rats and an in vitro model to research the protective effect of rhGPx4 on ISO-induced MI injury in rats and explore the antioxidant mechanism of rhGPx4.

## 2. Materials and Methods

### 2.1. Reagents

ISO, methyl thiazolyl tetrazolium (MTT), rhodamine (Rh123), and 4′,6-didiayl-2-phenylindole (DAPI) were purchased from Sigma-Aldrich (USA). Dulbecco's Modified Eagle's Medium (DMEM), fetal bovine serum (FBS), and 0.25% trypsin-EDTA were purchased from Gibco (USA). Dihydroethidine (DHE), Fluo-3 AM, ROS detection kit, and hematoxylin-eosin (H&E) staining Kit were purchased from Beyotime (China). The assay kits for lactate dehydrogenase (LDH), aspartate transaminase (AST), creatine kinase (CK), malondialdehyde (MDA), succinate dehydrogenase (SDH), and malate dehydrogenase (MDH) were purchased from Jiancheng (China). Cardiac troponin T (cTnT) ELISA Kit was purchased from Longton (China). In Situ Cell Death Detection Kit-POD was purchased from Roche (Switzerland). Annexin V-FITC/PI Apoptosis Detection Kit was purchased from BD (USA).

### 2.2. Preparation of rhGPx4

The rhGPx4 was derived from a previous study [13]. The prepared rhGPx4 was treated with Factor Xa Protease to remove His-tag. Then, rhGPx4 was confirmed by SDS-PAGE analysis as described previously [13].

### 2.3. Molecular Modeling of rhGPx4

The molecular model of rhGPx4 was the protein structure of high resolution structure of human GPx4 (PDB: 6ELW) [19]. The molecular model was observed using PyMOL (open-source software published by DeLano Scientific LLC).

### 2.4. Animals and Treatment

Male Sprague-Dawley (SD) rats (220-250 g) were provided by the Experimental Animal Center of Jilin University. All rats were acclimatized for at least one week before starting the study. Before and during the study, the animals were randomly group housed in cages and fed under a 12 h light/dark cycle at 22 ± 2°C and 60-70 % humidity with free access to sterile water and rodent food. Animal experiments were performed according to the Guide for the Care and Use of Laboratory Animals [United States National Institutes of Health (NIH)] and the Committee for the Care and Use of Laboratory Animals of Jilin University (Changchun, China). The study protocol was approved by the Ethics Committee of Jilin University.

**Figure 1:**
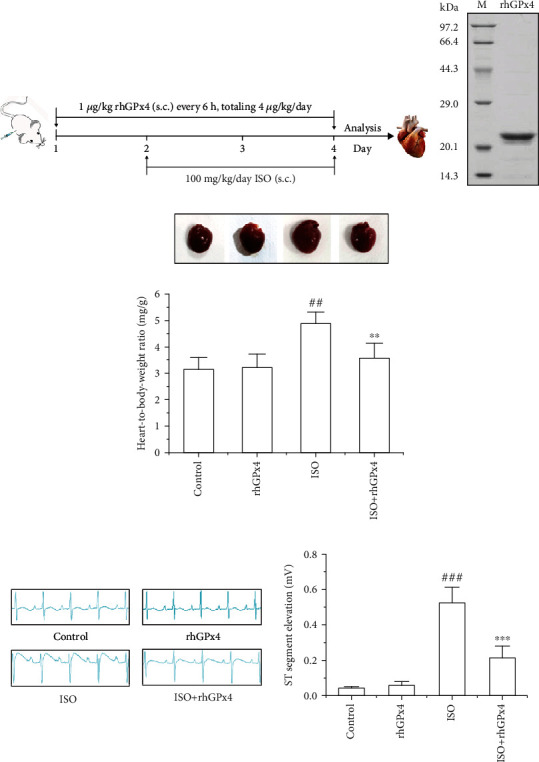
rhGPx4 prevents pathological changes of MI. (a) Schematic diagram of animal experiments. s.c. stands for subcutaneous injection. (b) SDS-PAGE analysis of rhGPx4 (21 kDa). M is the standard protein MW marker. (c) Representative images of cardiac hypertrophy and heart-to-body weight ratio in rats. (d) Representative images of ECG analysis and ST segment elevation changes in rats. Data represent the mean ± SD (*n* = 6). ^##^*P* < 0.01, ^###^*P* < 0.001 vs. control; ^∗∗^*P* < 0.01, ^∗∗∗^*P* < 0.001 vs. ISO.

After determining the optimal dose of rhGPx4 by preliminary experiments, all rats were randomly divided into 4 groups with 10 rats in each group. Group I (control): rats received only saline subcutaneously (s.c.) daily for a period of 3 days; group II (rhGPx4): rats received only 1 *μ*g/kg rhGPx4 (s.c.) every 6 h, totaling 4 *μ*g/kg/day for 3 days; group III (ISO): after the rats received saline for 1 days, the rats received 100 mg/kg/day ISO (s.c.) for 2 days; and group IV (ISO+rhGPx4): rats received 1 *μ*g/kg rhGPx4 (s.c.) every 6 h, totaling 4 *μ*g/kg/day for 3 days, and on the second and third day, the rats received 100 mg/kg/day ISO (s.c.) for 2 days. After ISO (s.c.) 24 h at last time, all rats were anesthetized by pentobarbital sodium (40 mg/kg, i.p.). Then, electrocardiograph (ECG) was recorded continually. After recording the ECG, blood was collected in the absence of anticoagulant, and serum was separated by centrifugation. After the rats were sacrificed, the heart tissue was excised and used for various studies. The schematic diagram of animal experiments was shown in Figure 1(a).

### 2.5. ECG Detection

The ECG patterns (Lead II) were recorded by Animal ECG Acquisition and Analysis System SP2006 (Ruanlong Biotechnology Co., Ltd., China). ECG recordings were made in anesthetized rats. The types of alterations (ST-segment elevation or depression) were recorded in rats.

### 2.6. Heart-to-Body-Weight Ratio

Determining the heart-to-body-weight ratio of rats was essential to research the effect of rhGPx4 on cardiac hypertrophy. After rats were sacrificed, the hearts were removed and washed with saline, and the weight of hearts was recorded by precision balance. The body weights of the rats were regularly monitored throughout the experiment.

### 2.7. Cardiac Serum Biomarker Assay

Serum samples were assayed to determine myocardial enzymes (LDH, CK, and AST) using commercial kits following manufacturer's instructions. cTnT was performed using a commercial ELISA kit following the manufacturer's instructions.

### 2.8. Myocardial DHE Staining

ROS generation in the heart tissue was detected by DHE staining [20]. Briefly, after their removal, the hearts were embedded in OCT compound and cut into 5 *μ*m-thick frozen slices. Frozen slices were stained with 10 *μ*M DHE at 37°C for 30 min and stained with 10 *μ*g/mL DAPI at 37°C for 10 min. The images were acquired by a fluorescence microscope (Olympus BX53, Japan) and analyzed by ImageJ 1.6 software.

### 2.9. Determination of Lipid Peroxidation

MDA was measured as a marker of oxidative stress in the serum and heart tissue [21]. Briefly, according to the heart tissue weight, physiological saline was added with the ratio of V(mL): W(g) = 9 : 1, and the heart tissue homogenate was obtained by the mechanical homogenization under the ice-water bath. The heart tissue homogenate and sera were reacted with thiobarbituric acid (TBA). The mixtures were heated in a water bath for 40 min at 95°C, and the absorbance of MDA was detected at 530 nm. The lipid peroxidation level was expressed as nanomole of MDA production per gram of myocardium and per milliliter of serum.

### 2.10. Histopathology Analysis

After the rats were sacrificed, the hearts were fixed in 4% paraformaldehyde, embedded in paraffin, and then cut into 5 *μ*m heart slices with a microtome (Leica RM2235, Germany). H&E staining was used to assess the morphology of the myocardium. The heart slices were examined with an optical microscope (Olympus CK21, Japan), and the micrographs were analyzed by two pathologists who did not know the treatment.

### 2.11. Measurement of Infarct Size

The cardiac infarct size was determined by triphenyltetrazolium chloride (TTC) staining [22]. Briefly, transverse cardiac slices of 2 mm thickness were incubated with 1% TTC stain for 20 min at 37°C, followed by fixation with 4% paraformaldehyde. Normal cardiac tissues (noninfarct region) appeared red, whereas abnormal cardiac tissues (infarct region) appeared pale grey. A camera was used to image the cardiac slices, and the infarct size was measured using ImageJ 1.6 software.

### 2.12. Transmission Electron Microscope (TEM) Detection

To observe the morphology of mitochondria in the myocardial tissue, TEM was performed on it. Briefly, the myocardial tissue was fixed with 2.5% glutaraldehyde and 1% osmium acid, dehydrated with ethanol, embedded in eponate 12 epoxy resin, and made into ultra-thin sections. Double staining with 3% uranyl acetate-lead citrate and filming with a TEM (FEI tecnai spirit, USA).

### 2.13. Mitochondrial Enzyme Detection

SDH and MDH are key enzymes involved in the mitochondrial tricarboxylic acid cycle, and their activity can reflect the function of mitochondria. Tissue samples were made into 10% tissue homogenate with physiological saline, and tissue homogenates were assayed to determine activity of mitochondrial enzyme using SDH and MDH commercial kits following manufacturer's instructions.

### 2.14. TUNEL Staining

TUNEL staining was used to detect DNA fragmentation for apoptosis [23]. Briefly, after dewaxing and hydrating the paraffin slices, they were digested with proteinase K at 37°C for 20 min, and then the TUNEL reaction mixtures were incubated at 37°C in the dark. Subsequently, the slices were covered with converter-POD at 37°C for 30 min, and then incubation with DAB for 3 min was used to visualize the reaction. Finally, the slices were counterstained with hematoxylin for 30 s, and positively stained cells were counted in six different fields per slice.

### 2.15. Cell Culture and Treatment

H9C2 cells were obtained from American Type Culture Collection (ATCC, USA) and cultured in high-glucose DMEM with 10% FBS in a 5% CO_2_ incubator at 37°C. In the experiment, we first determined the optimal dose of rhGPx4 by preliminary experiments, and then cell experiments were divided into 4 groups: group I (control): H9C2 cells were treated with PBS (the same amount as ISO) for 24 h; group II (rhGPx4): H9C2 cells were treated with 0.3 *μ*g/mL rhGPx4 for 24 h; group III (ISO): H9C2 cells were treated with 50 *μ*M ISO for 24 h; and group IV (ISO+rhGPx4): H9C2 cells were pretreated with 0.3 *μ*g/mL rhGPx4 for 1 h and then incubated with 50 *μ*M ISO for 24 h.

### 2.16. Cell Damage Analysis

Cell viability was evaluated by the MTT assay. Briefly, H9C2 cells were seeded in 96-well plates. After the treatment, the cells were incubated with MTT (final concentration 0.5 mg/mL) for 4 h. The formazan precipitates were dissolved in 200 *μ*L of DMSO, and the absorbance was detected at 490 nm with a microplate reader (Bio-TEK, USA). The LDH release rate of cells was measured using an LDH activity assay kit following the manufacturer's instructions. Briefly, H9C2 cells were seeded in 96-well plates. After treatment, culture media were collected and transferred to another 96-well plates. LDH reaction mixes were added to each well, and the plates were incubated for 30min at room temperature (RT). The absorbance was detected at 450 nm.

### 2.17. Measurement of ROS

Cellular ROS contents were evaluated by DCFH-DA and DHE [24]. Briefly, H9C2 cells were seeded in 6-well plates. After treatment, cells were stained with 10 *μ*M DCFH-DA and 5 *μ*M DHE at 37°C for 30 min in the dark, respectively. Images were obtained using a fluorescence microscope (Olympus CKX53, Japan), and the relative fluorescence intensity was measured using ImageJ 1.6 software.

### 2.18. Mitochondrial Membrane Potential Analysis

Mitochondrial membrane potential in H9C2 cells were measured using the Rh123. Briefly, after the treatment, cells were stained with 10 *μ*g/mL Rh123 for 30 min at 37°C in the dark. The images were obtained using a fluorescence microscope (Olympus CKX53, Japan), and the relative fluorescence was measured using ImageJ 1.6 software.

### 2.19. Detection of Ca^2+^

Cellular Ca^2+^ contents were evaluated by Fluo-3 AM. Briefly, H9C2 cells were seeded in 6-well plates. After treatment, cells were stained with 5 *μ*M Fluo-3 AM at 37°C for 60 min in the dark. Fluorescence intensity of Fluo-3 was detected using a flow cytometer (BD Accuri C6, USA).

### 2.20. Annexin V-FITC/PI Apoptosis Assay

Annexin V-FITC/PI staining was used to detect cell apoptosis. Briefly, H9C2 cells were seeded in 6-well plates. After treatment, the cells were harvested and stained with 5 *μ*L of Annexin V-FITC and 10 *μ*L of PI for 15 min at RT in the dark. Apoptotic cells were detected using a flow cytometer (BD Accuri C6, USA).

### 2.21. Western Blot Analysis

After treatment, nuclear proteins and cytoplasmic proteins were extracted from tissues and cells using the Nuclear and Cytoplasmic Protein Extraction Kit (Beyotime, China) containing 1% PMSF and quantified using a bicinchoninic acid (BCA) protein kit. Then, 40 *μ*g of protein was separated by SDS-PAGE and electrotransferred onto PVDF membranes (Millipore, USA). The PVDF membranes were blocked in 1% BSA for 1 h and incubated overnight at 4°C with primary antibodies against the following: Nrf2 (1 : 500; cat. no. sc-722; Santa Cruz), Lamin B (1 : 1000; cat. no. 12586; Cell Signaling Technology), HO-1 (1 : 10000; cat. no. ab68477; Abcam), Bax (1 : 1000; cat. no. 2772; Cell Signaling Technology), Bcl-2 ( 1: 1500; cat. no. ab196495; Abcam), cleaved caspase-3 (1 : 1000; cat. no. 9661; Cell Signaling Technology), and *β*-actin (1 : 5000; cat. no. AF5003; Beyotime). Then, the PVDF membranes were incubated with the corresponding goat anti-rabbit secondary antibodies (1 : 1000; cat. no. A0208; Beyotime) at RT for 1 h. The protein bands in the PVDF membranes were detected by an ECL detection kit and exposed using the chemiluminescent gel imaging system (BIO-RAD, USA). The proteins were normalized to lamin B and *β*-actin.

### 2.22. Statistical Analysis

SPSS 19.0 software (SPSS Inc., USA) was used to analyze the results. Data were expressed as the means ± standard deviation (SD), and significance was measured by using one-way analysis of variance (ANOVA). Student's *t*-test was used to determine the differences between groups. The probability values of *P* < 0.05 were considered significant.

## 3. Results

### 3.1. rhGPx4 Inhibits ISO-Induced Cardiac Hypertrophy

Firstly, the expressed and purified rhGPx4 was identified by SDS-PAGE. As shown in Figure 1(b), there was a single electrophoresis band at 21 kDa for rhGPx4, which indicated that rhGPx4 was expressed and purified with high purity. Then, cardiac hypertrophy was analyzed by the heart-to-body-weight ratio of rats. As shown in Figure 1(c), compared with that of control group, the heart-to-body-weight ratio of the ISO group notably increased. Compared with that in the ISO group, the heart-to-body-weight ratio in the ISO+rhGPx4 group was diminished. This result suggested that rhGPx4 could inhibit ISO-induced cardiac hypertrophy.

### 3.2. rhGPx4 Prevents ISO-Induced Abnormal ECG

As shown in Figure 1(d), both the control group and rhGPx4 group showed normal ECG. Compared with that of the control group, the ECG of the ISO group showed obvious ST segment elevation. Compared with that of the ISO group, the ECG of the ISO+rhGPx4 group showed a decrease in the ST segment. This result suggested that rhGPx4 could prevent ISO-induced abnormal changes in ECG.

### 3.3. rhGPx4 Ameliorates ISO-Induced Abnormal Biochemical Indicators

**Figure 2:**
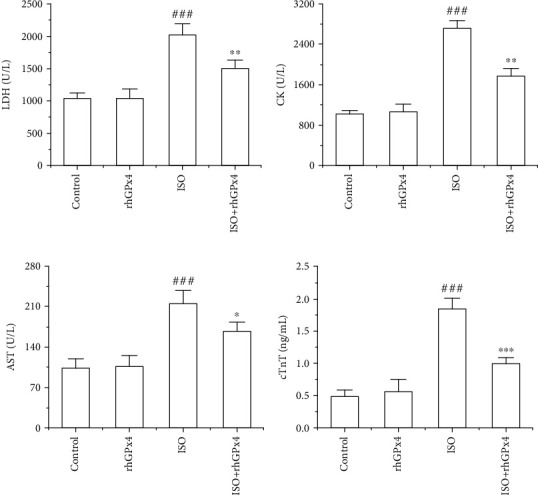
rhGPx4 improves abnormal serum biochemical indicators. The activity of (a) LDH, (b) CK, and (c) AST in rat serum. (d) The level of cTnT in rat serum. Data represent the mean ± SD (*n* = 6). ^###^*P* < 0.001 vs. control; ^∗^*P* < 0.05, ^∗∗^*P* < 0.01, ^∗∗∗^*P* < 0.001 vs. ISO.

As shown in Figure 2, compared with those of the control group, the activity of LDH, CK, AST, and the level of cTnT in the serum of the ISO group significantly increased. Compared with those of the ISO group, the activity of LDH, CK, AST, and the level of cTnT in the serum of the ISO+rhGPx4 group was reduced. This result suggested that rhGPx4 could inhibit ISO-induced abnormal elevation of serum biochemical indicators.

### 3.4. rhGPx4 Prevents ISO-Induced Pathological Damage

**Figure 3:**
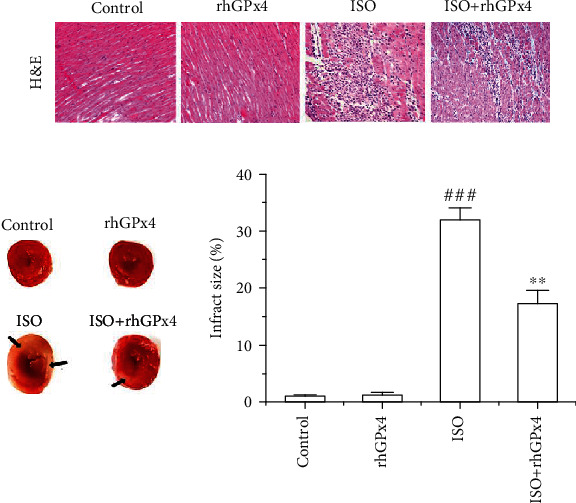
rhGPx4 prevents pathological damage of MI. (a) Representative images of H&E staining (optical microscope, ×200). (b) Representative images of TTC staining of the heart tissue and the percentage of the infarct size. The black arrow indicates the location of MI, and the percentage of the infarct size in the control group was set to 1%. Data represent the mean ± SD (*n* = 3). ^###^*P* < 0.001 vs. control; ^∗∗^*P* < 0.01 vs. ISO.

The results of H&E staining are shown in Figure 3(a), and normal architectures were observed in the control group and rhGPx4 group. Compared with the control group, the ISO group showed disordered arrangement of myocardial cells, interstitial edema, and extensive inflammatory cell infiltration. However, compared with that in the ISO group, the degree of myocardial injury was greatly reduced in the ISO+rhGPx4 group. Additionally, we analyzed the area of myocardial infarction in rats by TTC staining. As shown in Figure 3(b), the control group and rhGPx4 group showed myocardial tissue without any infarction. Compared with the control group, the ISO group showed infarcted heart tissues, which failed to stain with TTC. Compared with that of the ISO group, the infarct area of the heart tissue was reduced in the ISO+rhGPx4 group. Overall, these results suggested that rhGPx4 could ameliorate ISO-induced pathological changes and reduce the area of myocardial infarction.

### 3.5. rhGPx4 Reduces ISO-Induced Oxidative Stress Injury

**Figure 4:**
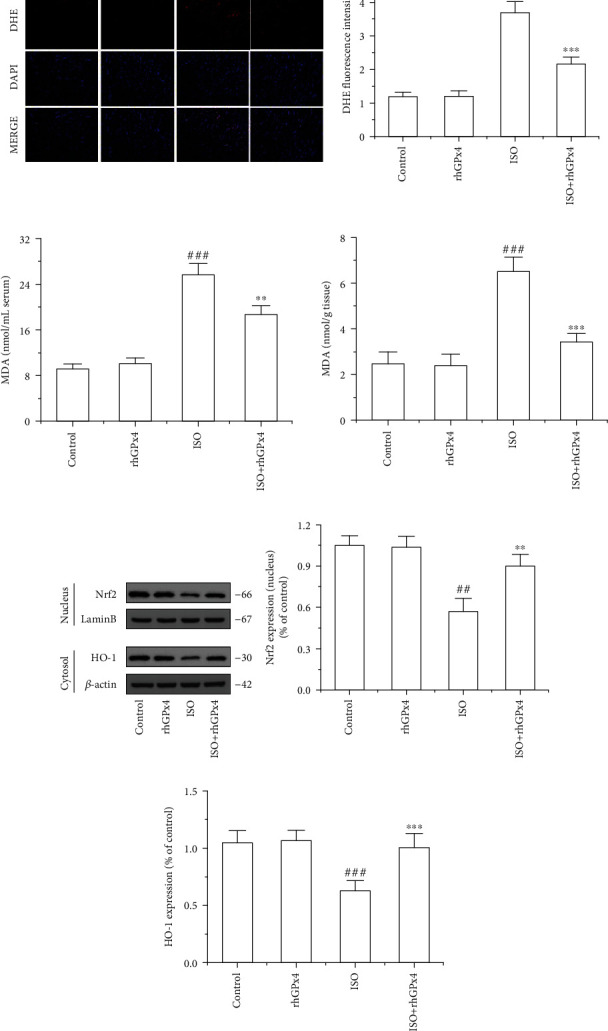
rhGPx4 reduces oxidative stress injury in heart tissue. (a) Representative images of DHE staining of the heart tissue (fluorescence microscope, ×200) and DHE fluorescence intensity. (b) The effects of rhGPx4 on MDA levels in the serum and heart tissue. (c) Representative western blot bands of Nrf2, Lamin B, HO-1, and *β*-actin. The expression levels of (d) Nrf2 (Nucleus) and (e) HO-1 in the heart tissue. Data represent the mean ± SD (*n* = 3). ^##^*P* < 0.01, ^###^*P* < 0.001 vs. control; ^∗∗^*P* < 0.01, ^∗∗∗^*P* < 0.001 vs. ISO.

**Figure 5:**
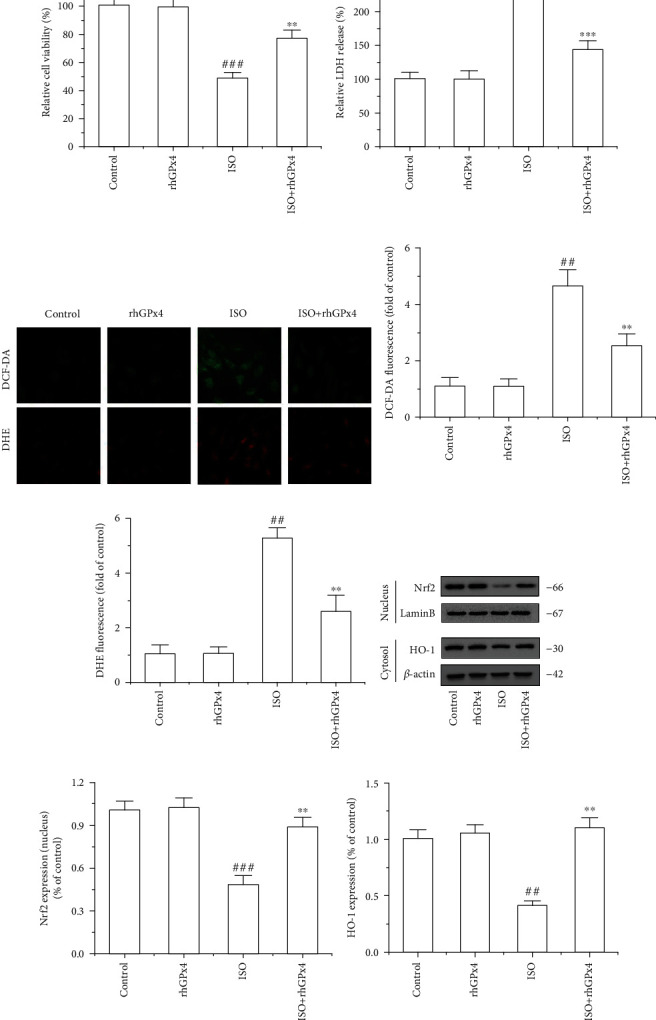
rhGPx4 reduces oxidative stress injury in H9C2 cells. (a) The viability of H9C2 cells was detected by the MTT assay. (b) H9C2 cell injury was assessed by LDH release. Data represent the mean ± SD (*n* = 6). (c) Representative images of DCFH-DA and DHE staining of H9C2 cells (fluorescence microscope, ×200). Fluorescence intensity of (d) DCF-DA and (e) DHE in H9C2 cells. (f) Representative western blot bands of Nrf2, Lamin B, HO-1, and *β*-actin. The expression levels of (g) Nrf2 (nucleus) and (h) HO-1 in H9C2 cells. Data represent the mean ± SD (*n* = 3). ^##^*P* < 0.01, ^###^*P* < 0.001 vs. control; ^∗∗^*P* < 0.01, ^∗∗∗^*P* < 0.001 vs. ISO.

The production of ROS in the heart tissue was observed by DHE staining. As shown in Figure 4(a), compared with that in the control group, the fluorescence intensity of DHE in the ISO group was substantially increased. Compared with that in the ISO group, the fluorescence intensity of DHE in the ISO+rhGPx4 group was weaker. To determine the lipid peroxidation, MDA levels were measured in the serum and heart tissue. As shown in Figure 4(b), compared with those in the control group, MDA levels of the ISO group significantly increased. However, compared with those of the ISO group, the MDA levels of the ISO+rhGPx4 group reduced. This suggested that rhGPx4 could reduce ISO-induced cardiac oxidative stress injury. Then, we explored whether rhGPx4 could affect the level of NF-E2-related factor 2 (Nrf2) and heme oxygenase-1 (HO-1) in the heart tissue. As shown in Figures 4(c)–(e), compared with the control group, the ISO group showed decreased expression of Nrf2 in the nucleus and the decreased expression of HO-1 in the cytoplasm. This suggested that the oxidative stress caused by ISO could consume oxidative stress regulator Nrf2 in the nucleus and the endogenous antioxidant enzyme HO-1 in the cytoplasm. However, compared with that in the ISO group, in the ISO+rhGPx4 group, the expression of Nrf2 in the nucleus and the expression of HO-1 in the cytoplasm basically maintained to normal. This suggested that rhGPx4 could keep the normal expression of oxidative stress regulator Nrf2 in the nucleus and endogenous antioxidant enzyme HO-1 in the cytoplasm by eliminating ROS. To further verify the antioxidant effect of rhGPx4, ISO-induced H9C2 cells were selected as a cell model in vitro. As shown in Figure 5(a), compared with that of the control group, the cell viability of the ISO group significantly decreased, and compared with that of the ISO group, the cell viability of the ISO+rhGPx4 group increased. The LDH release rate was used to analyze the cell injury. As shown in Figure 5(b), compared with that of the control group, the LDH release rate of the ISO group notably increased, and compared with that of the ISO group, the LDH release rate of the ISO+rhGPx4 group was reduced. Additionally, we detected the generation of ROS in H9C2 cells by DCFH-DA and DHE staining. As shown in Figures 5(c)–(e), compared with that in the control group, the fluorescence intensity of DCF and DHE in the ISO group significantly increased, and compared with the ISO group, the fluorescence intensity of DCF and DHE in the ISO+rhGPx4 group weakened. This suggested that rhGPx4 could inhibit ISO-induced oxidative stress injury in H9C2 cells. The expression of NF-E2-related factor 2 (Nrf2) and heme oxygenase-1 (HO-1) in H9C2 cells were shown in Figures 5(f)–(h). The results were in accordance with in vivo studies. Overall, these results indicated that rhGPx4 could not only inhibit lipid peroxidation but also maintain a normal level of endogenous antioxidant enzymes by eliminating ROS.

### 3.6. rhGPx4 Protects Cardiomyocyte Mitochondrial Function

**Figure 6:**
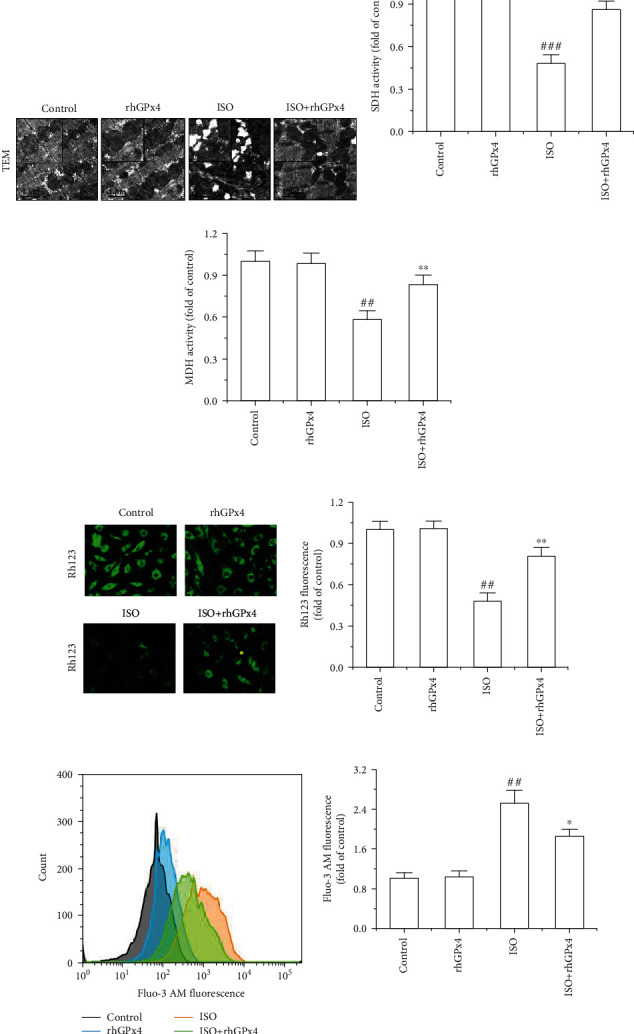
rhGPx4 prevents cardiomyocyte mitochondrial injury. (a) Representative images of TEM of the cardiomyocyte mitochondrial. (b) SDH. (c) MDH activity of the heart tissue. (d) Representative images of Rh123 staining of H9C2 cells (fluorescence microscope, ×200) and fluorescence intensity of Rh123. (e) Representative images of Fluo-3 AM staining of H9C2 cells and fluorescence intensity of Fluo-3 AM. Data represent the mean ± SD (*n* = 3). ^##^*P* < 0.01, ^###^*P* < 0.001 vs. control; ^∗^*P* < 0.05, ^∗∗^*P* < 0.01 vs. ISO.

Ultrastructure of cardiomyocyte mitochondria was detected by TEM. As shown in Figure 6(a), normal ultrastructures of cardiomyocyte mitochondria were observed in the control group and rhGPx4 group. Compared with the control group, the ISO group showed that the mitochondria were swollen, and the cristae were broken and dissolved into vacuoles. However, compared with that in the ISO group, the degree of myocardial injury was greatly reduced in the ISO+rhGPx4 group. Activities of mitochondrial enzymes were measured in the heart tissue. As shown in Figures 6(b) and 6(c), compared with those of the control group, activity of SDH and MDH of the ISO group significantly reduced. However, compared with those of the ISO group, activity of SDH and MDH of the ISO+rhGPx4 group increased. This suggested that rhGPx4 could protect the mitochondrial structure and maintain mitochondrial function in cardiomyocytes. Additionally, to further verify the protective effect of rhGPx4 on mitochondria, we used Rh123 staining to analyze the mitochondrial membrane potential in H9C2 cells. As shown in Figure 6(d), compared with that in the control group, the fluorescence intensity of Rh123 in the ISO group significantly weakened, and compared with the ISO group, the fluorescence intensity of Rh123 in the ISO+rhGPx4 group increased. Intracellular Ca^2+^ overload can cause mitochondrial dysfunction. The level of Ca^2+^ in H9C2 cells was observed by Fluo-3 AM staining. As shown in Figure 6(e), compared with that in the control group, the fluorescence intensity of Fluo-3 AM in the ISO group significantly increased, and compared with the ISO group, the fluorescence intensity of Fluo-3 AM in the ISO+rhGPx4 group weakened. Overall, these results indicated that rhGPx4 could prevent Ca^2+^ overload and protect the mitochondrial structure by eliminating ROS, thereby preventing mitochondrial dysfunction of cardiomyocytes.

### 3.7. rhGPx4 Inhibits ISO-Induced Cardiomyocyte Apoptosis through a Mitochondria-Dependent Pathway

**Figure 7:**
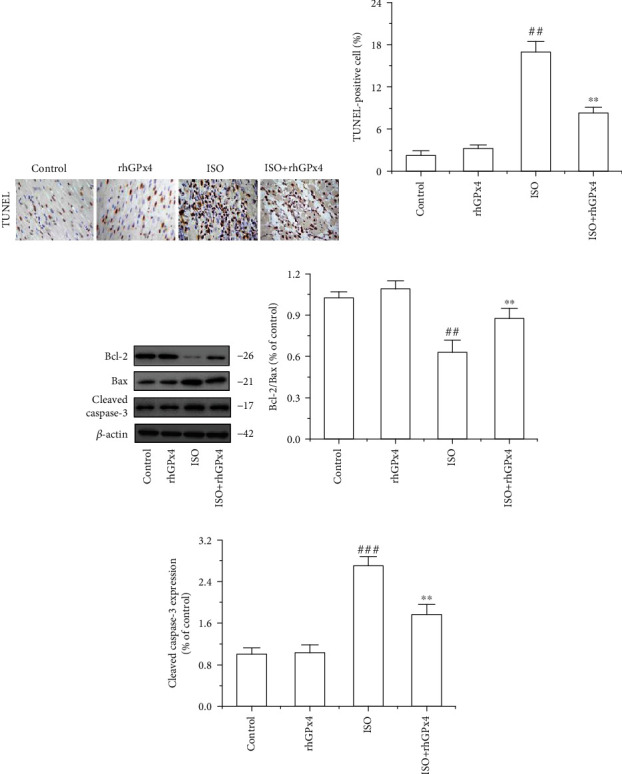
rhGPx4 inhibits cardiomyocyte apoptosis in the heart tissue. (a) Representative images of TUNEL staining of the heart tissue (optical microscope, ×400). The apoptotic cardiomyocyte nucleus appears brown, and normal nuclei appear blue. (b) Ratio of TUNEL-positive cells (%). (c) Representative western blot bands of Bcl-2, Bax, Cleaved caspase-3, and *β*-actin. (d) The ratio of the Bcl-2/Bax expression in the heart tissue. (e) The expression levels of cleaved caspase-3 in the heart tissue. Data represent the mean ± SD (*n* = 3). ^##^*P* < 0.01, ^###^*P* < 0.001 vs. control; ^∗∗^*P* < 0.01 vs. ISO.

**Figure 8:**
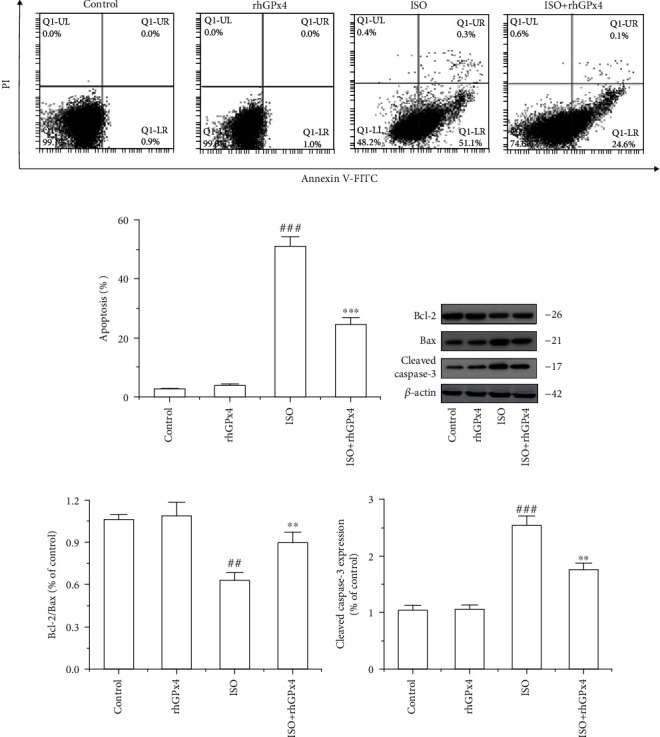
rhGPx4 inhibits apoptosis in H9C2 cells. (a) Representative images of Annexin-V/PI staining of H9C2 cells. (b) The apoptotic rates of H9C2 cells. The apoptosis rate is the sum of the early and late apoptosis rates. (c) Representative western blot bands of Bcl-2, Bax, Cleaved caspase-3, and *β*-actin. (d) The ratio of the Bcl-2/Bax protein expression in H9C2 cells. (e) The expression levels of cleaved caspase-3 in H9C2 cells. Data represent the mean ± SD (*n* = 3). ^##^*P* < 0.01, ^###^*P* < 0.001 vs. control; ^∗∗^*P* < 0.01, ^∗∗∗^*P* < 0.001 vs. ISO.

We used TUNEL staining to detect cardiomyocyte apoptosis in the heart tissue. As shown in Figures 7(a) and 7(b), compared with that in the control group, the ratio of TUNEL-positive cells in the ISO group was greatly increased. However, compared with that in the ISO group, the ratio of TUNEL-positive cells in the ISO+rhGPx4 group was decreased. The expression of Bax, Bcl-2, and cleaved caspase-3 in the heart tissue was detected by western blotting. As shown in Figures 7(c)–(e), compared with that in the control group, the ratio of the Bcl-2/Bax expression of in the ISO group significantly decreased, and the expression of cleaved caspase-3 increased. Compared with that in the ISO group, the ratio of the Bcl-2/Bax expression in the ISO+rhGPx4 group increased, and the expression of cleaved caspase-3 decreased. Additionally, to further verify the above effect of rhGPx4 on cell apoptosis, we used Annexin V-FITC/PI staining to analyze the apoptosis of H9C2 cells. As shown in Figures 8(a) and 8(b), compared with those of the control group, the apoptosis rates of the ISO group notably increased. Compared with those of the ISO group, the apoptosis rates of the ISO+rhGPx4 group were reduced. The expression of Bax, Bcl-2, and cleaved caspase-3 in H9C2 cells was detected by western blotting. As shown in Figures 8(c)–(e), compared with that in the control group, the ratio of the Bcl-2/Bax expression in the ISO group dramatically decreased, and the expression of cleaved caspase-3 increased. Compared with that in the ISO group, the ratio of the Bcl-2/Bax expression in the ISO+rhGPx4 group increased, and the expression of cleaved-caspase-3 decreased. In short, these results suggested that rhGPx4 could inhibit ISO-induced cardiomyocyte apoptosis through a mitochondria-dependent pathway.

## 4. Discussion

IHD is a cardiovascular disease caused by myocardial ischemia and hypoxia. Conclusive evidence has demonstrated that oxidative stress caused by excess ROS plays a vital role in the occurrence and development of MI [25]. Therefore, antioxidative stress may be an effective strategy to prevent IHD. In a previous study, we expressed rhGPx4 in amber-less *E. coli* using a novel chimeric tRNA^UTuT6^ [13]. In the present study, rhGPx4, a recombinant antioxidant enzyme, exerted a strong antioxidant effect on ISO-induced MI in rats. ECG abnormalities, including ST-segment elevation, are one of the important signs of cardiomyocyte membrane injury [26]. Recent studies have confirmed that GPx4 can protect cell membranes from oxidative stress injury and work with alpha tocopherol to inhibit cell lipid peroxidation [27]. In this study, we found that rhGPx4 could prevent abnormalities in the ST segment of the ECG. This meant that rhGPx4 also had a protective effect on cell membranes and prevented lipid peroxidation. Because the permeability of cardiomyocyte membranes increases during MI, the abnormal increase in LDH, CK, and AST in the blood can reflect damage to the cardiomyocyte membranes. We found that rhGPx4 could inhibit the abnormal increase in myocardial injury enzymes, which again proved that rhGPx4 was a cell membrane protector.

**Figure 9:**
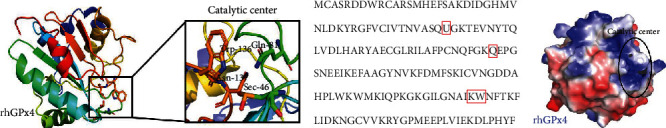
Amino acid sequence and structure of rhGPx4. (a) Overall structure and catalytic center of rhGPx4 (PDB: 6ELW). The catalytic tetrads consisting of Sec, Gln, Trp, and Asn are labeled. (b) Amino acid sequence of rhGPx4. The catalytic tetrads are marked. (c) Electrostatic potential surface representation of rhGPx4. The catalytic center is circled. Red indicates a negative electrostatic potential, and blue indicates a positive electrostatic potential. Cartoon representations were generated in PyMol.

**Figure 10:**
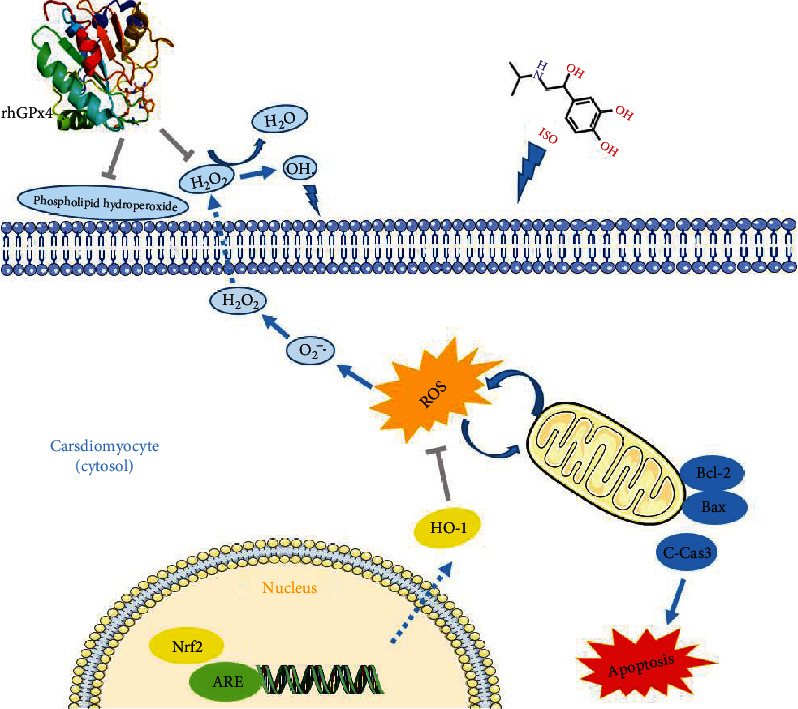
Schematic illustration of the mechanism of rhGPx4 against oxidative stress injury and apoptosis.

To further study the antioxidant effect of rhGPx4, we analyzed the structural characteristics of rhGPx4. In the GPx family, GPx4 has a monomer structure. In previous studies, we found that rhGPx4 was also a monomeric selenoprotein [13]. In addition, evidence has indicated that the catalytic center of GPx contains a catalytic tetrad, which plays a major role in the catalytic reaction [28]. In the molecular model of rhGPx4, we observed that the catalytic tetrad of rhGPx4 was located on the surface of the enzyme, and that a large number of positive charges were distributed around it (Figure 9). In the structure of GPx, the positively charged area of the catalytic center can promote the nucleophilic attack of GPx on hydrogen peroxide through electrostatic attraction [29]. Moreover, GPx4 can promote the reaction with lipid hydroperoxide through specific electrostatic interactions [30]. Therefore, with the unique molecular structure of the enzyme, rhGPx4 can catalyze not only small molecular hydroperoxides but also complex lipid hydroperoxides, thereby protecting cells from oxidative stress injury. In addition, GPx4 has a variety of reducing substrates [31]. Since rhGPx4 is a water-soluble recombinant protein, it cannot pass through the phospholipid bilayer of the cell membrane. Nevertheless, rhGPx4 as a recombinant antioxidant enzyme cannot only catalyze H_2_O_2_ into water in the extracellular environment but also act as a cell membrane protector to inhibit oxidative lipidation of on cell membranes (Figure 10).

Nrf2 is an important redox regulator in cells and is considered a key factor in resisting oxidative stress [32]. Increasing evidence has revealed that Nrf2 is important in the process of preventing cardiac oxidative stress injury [33–35], and the endogenous antioxidant enzyme HO-1 also has the effect of inhibiting oxidative stress injury in cardiomyocytes [36]. In this study, rhGPx4 could keep a normal level of oxidative stress regulator Nrf2 in the nucleus and endogenous antioxidant enzyme HO-1 in the cytoplasm by eliminating ROS. This meant that during oxidative stress of cardiomyocytes, rhGPx4 not only could inhibit lipid peroxidation by eliminating ROS but also maintain a normal level of endogenous antioxidant enzymes by eliminating ROS, thereby preventing the oxidative stress injury of cardiomyocytes. This also further proved the high efficiency of rhGPx4 in eliminating ROS.

Mitochondria are important organelles that produce energy, and they are widely distributed in cardiomyocytes. When oxidative stress occurs in the heart, mitochondria are vulnerable to ROS attack, which makes mitochondria dysfunction, thus affecting the occurrence and development of MI [37, 38]. Moreover, sarcoplasmic reticulum is the calcium storehouse in myocardial cells. Excessive ROS will destroy the sarcoplasmic reticulum membrane and cause a large amount of Ca^2+^ to leak into the cytoplasm, causing intracellular Ca^2+^ overload. Excessive Ca^2+^ will destroy the function of mitochondria and promote cardiomyocyte apoptosis. It is reported that reducing Ca^2+^ overload and reducing ROS can reduce MI injury [39]. In this study, rhGPx4 could eliminate ROS, protect mitochondrial structure from oxidative stress injury, and inhibit Ca^2+^ overload, thereby preventing cardiomyocyte mitochondrial dysfunction. The Bcl-2 family plays a key role in the process of cell apoptosis, and decreased expression ratio of Bcl-2 to Bax is one of the causes of cardiomyocyte apoptosis [40]. Bax can induce cytochrome C to enter the cytoplasm to form an apoptotic complex. The apoptotic complex drives caspase-9 activation, which then activates caspase-3 and finally initiates apoptosis [41, 42]. In this study, rhGPx4 increased the expression ratio of Bcl-2/Bax and inhibited the expression of cleaved caspase-3. This meant that rhGPx4 could inhibit cardiomyocyte apoptosis through a mitochondria-dependent pathway.

## 5. Conclusion

In summary, rhGPx4, a recombinant antioxidant enzyme, exerts cardioprotective effects on ISO-induced MI injury, which can reduce cardiac oxidative stress injury and improve MI. The mechanism of action of rhGPx4 involves not only the inhibition of lipid peroxidation by eliminating ROS but also keeping a normal level of endogenous antioxidant enzymes by eliminating ROS, thereby preventing oxidative stress injury in cardiomyocytes. Moreover, rhGPx4 can also protect mitochondrial function and inhibit cardiomyocyte apoptosis through the mitochondrial-dependent pathway. Based on these findings, rhGPx4 may be a potential cardioprotective drug for IHD.

## Data Availability

The original data used to support the findings of this study are available from the corresponding author upon request.
